# Breastfeeding has no protective effects on the development of coronary artery lesions in Kawasaki disease: a retrospective cohort study

**DOI:** 10.1186/s12887-022-03422-y

**Published:** 2022-06-20

**Authors:** Hongli Wang, Yunjia Tang, Wenhua Yan, Qiuqin Xu, Xuan Li, Weiguo Qian

**Affiliations:** 1 Department of Cardiology, Children’s Hospital of Soochow University, No. 92, Zhongnan Street, Suzhou, China; 2 Department of Cardiology, Qilu Children’s Hospital of Shandong University, Jinan, China

**Keywords:** Breastfeeding, Child, Coronary Artery Lesions, Mucocutaneous Lymph Node Syndrome

## Abstract

**Background:**

Kawasaki disease (KD) is a self-limiting vasculitis with an unknown etiology. It has been reported that breastfeeding has a potential protective effect on KD development. However, whether breastfeeding has an effect on the development of coronary artery lesions (CALs) remains unclear.

**Methods:**

We retrospectively reviewed the medical records of patients with the main diagnosis of KD hospitalized in our hospital from May 2017 to November 2018. Standardized telephone interviews were carried out to obtain feeding practices before KD was onset.

**Results:**

Two hundred and ninety-three (51.6%) were exclusively breastfed, 223 (39.3%) were partially breastfed and 52 (9.2%) were formula fed. There were no significant differences in the characteristics regarding age, gender, incomplete KD, intravenous immunoglobulin (IVIG) resistance, and the laboratory variables among the three groups. With formula feeding as a reference, patients exclusively breastfed and partially breastfed seemed to have a higher incidence of CALs, even after adjusting confounders, but were not statistically significant. After grouping patients who were older than six months into formula feeding, partial breastfeeding for < 2 months, partial breastfeeding for ≥ 2 and < 4 months, partial breastfeeding for ≥ 4 and < 6 months and exclusively breastfeeding based on the length of breastfeeding, the results remained the same (*P* > 0.05).

**Conclusions:**

Breastfeeding has no protective effect on the development of CALs in KD.

**Supplementary Information:**

The online version contains supplementary material available at 10.1186/s12887-022-03422-y.

## Introduction

Kawasaki disease (KD) is a self-limiting vasculitis, that predominantly occurs in children under five years old. It is the main contributor to acquired heart diseases in developed countries due to its cardiac complications [1]. However, its specific causes remain to be elucidated despite years of research. Existing researches have hypothesized that KD is a consequence of an abnormal immunological response triggered by undefined infectious agents in genetically susceptible individuals [2, 3].

Breastmilk contains abundant active immune factors, which help to establish the mature immune system of infants. There is a great interest in the association between breastfeeding and immune diseases [4–6]. KD is one of the most commonly seen immune vasculitis in children, and whether its disease onset was associated with feeding practices made us confused. In 2016, Japanese researchers conducted a nationwide survey that proved the protective effect of breastfeeding in the development of KD for the first time [7]. However, whether breastfeeding has an effect on the development of coronary artery lesions (CALs) remains unclear. There were few studies carried out [8, 9]. In the present study, we tried to investigate the effects of feeding practices, as well as the length of breastfeeding on the development of CALs.

## Methods

### Study participants

We retrospectively reviewed the medical records of patients with the main diagnosis of KD hospitalized in Children’s Hospital of Soochow University from May 2017 to November 2018. The institutional review board of Children’s Hospital of Soochow University approved this study (No: 2017LW008) and waived the requirement for informed consent because of the retrospective nature of the study.

**Fig. 1:**
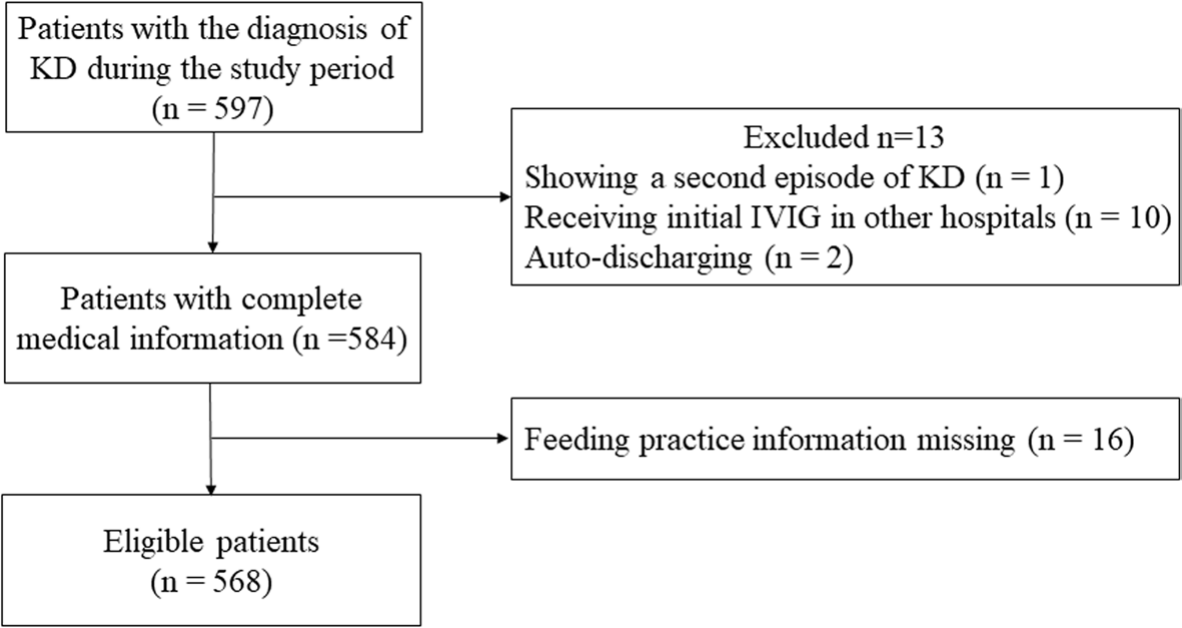
Study flow diagram. KD, Kawasaki disease

A total of 597 patients were initially diagnosed with KD and treated in our hospital during this study period. Thirteen patients were excluded, including one patient who showed a second episode of KD, one who did not receive initial intravenous immunoglobulin (IVIG) treatment due to defervescence before IVIG, 10 who received initial IVIG treatment in other hospitals prior to admission, and two who were auto-discharged with missing echocardiographic reports. Besides, 16 patients couldn’t be reached by telephone or caregivers couldn’t recall the feeding practices. As a result, 568 patients were enrolled in the study (Fig. 1).

### Definition

KD was diagnosed when a patient was febrile for ≥ five days, together with more than four of the following five characteristics: 1. Rash, 2. Bilateral conjunctive injection, 3. Cervical lymphadenopathy, 4. Changes of the extremities, 5. Oral mucosal changes [1]. Incomplete KD (iKD) was diagnosed in a patient who had two or three compatible clinical characteristics when the other possible causes of fever were excluded. A total of 2 g/kg IVIG in a single dose together with 30–50 mg/kg aspirin was administered as soon as possible after diagnosis in the febrile patients. For patients who were afebrile for 3–4 days, aspirin was then reduced to 3–5 mg/kg/day until the patients showed no evidence of CALs by 6 to 8 weeks after the onset of illness. Delayed IVIG treatment was defined as IVIG initiation after the 10^th^ day of illness. We calculated the z score of the left main coronary artery, left circumflex artery, left descending artery, and right coronary artery based on the method reported [10]. CALs were defined when the z score of any coronary artery ≥ 2.5; and/or the clearly irregular appearance of the intima. IVIG resistance was defined as persistent or recurrent fever > 38.0℃ lasting for more than 36 h [1]. A second dose of 2 g/kg IVIG was administered to these patients. Additional intravenous methylprednisolone of 2–4 mg/kg/day was also administered, which was switched to oral dose after defervescence and was tapered over 2 weeks. Exclusive breastfeeding was defined as totally breastfed while formula feeding was fed with formula milk when patients were under six months. Partial breastfeeding was intermediate. If a patient was now aged under six months and was breastfed since born, he was also defined as exclusive breastfeeding. Patients older than six months were included in further analysis to investigate the relationships between breastfeeding duration and the outcomes. Patients were grouped into five groups according to the length of breastfeeding, they were never breastfed (formula feeding), breastfed for < 2 months, breastfed for ≥ 2 and < 4 months, breastfed for ≥ 4 and < 6 months, and ≥ 6 months.

### Data collection

Standardized telephone interviews were carried out with guardians or caregivers of each patient to obtain feeding practices before KD was onset, as well as the length of breastfeeding. The laboratory variables and birth factors regarding parity, singleton or multiple births, preterm were reviewed from the medical records. All blood samples were obtained within the first 24 h on admission and after defervescence for more than 72 h, or as appropriate. An echocardiogram was carried out before the initial IVIG and was repeated before discharge. In patients with severe abnormity, it was repeated as appropriate. We chose the largest coronary internal lumen dimension for further analysis. The details of missing data are summarized in Additional file 1. More than 20% of the missing data were removed from our analysis, and the remaining missing data were obtained with the multiple imputation method.

### Statistical analyses

Statistical analyses were conducted using R for Windows (Version 4.0.4). Data were expressed as mean ± standard deviation (SD), median with quartiles, or number with percentage as appropriate. Descriptive statistics were performed on demographic characteristics. Parametric or nonparametric comparative tests for continuous data and χ2 test or Fisher exact test for categorical data were used to compare variables between groups. One-way ANOVA test or Kruskal–Wallis H test was used to compare continuous data among the three groups as appropriate. Bonferroni test was used to compare every two groups among the three groups based on the variance. Log-rank test for univariate analysis was performed to explore the risk factors for CALs on admission. The covariates, when added to the Cox regression model, changed the matched hazard ratio (HR) by at least 10% and were selected for adjustments. The variance inflation factor (VIF) method was used to examine multicollinearity of the covariances, and VIF ≥ 5 suggested multicollinearity. Feeding practices were chosen as categorical variables. In order to clearly describe the effect of breastfeeding on the outcomes. Three different models were established. Model 1 was a crude model. Model 2 adjusted for sex, delayed IVIG treatment, iKD, and IVIG resistance. Model 3 adjusted for confounders in model 2, plus singleton, prematurity, white blood cells (WBC), hematocrit, platelet, and alanine aminotransferase (ALT). We included IVIG resistance, sex, and delayed IVIG treatment into the Cox regression models because they were widely considered as the risk factors for CALs. Subgroup analyses regarding ages (< 1 yr, ≥ 1 and < 3 yrs, and ≥ 3 yrs) were carried out to analyze the associations between feeding practices and CALs. We included patients with complete medical data and did the statistics as a sensitivity analysis. Two-tailed *P* < 0.05 was considered statistically significant.

## Results

There were 355 males and 213 females included in the study. iKD accounted for 121 (21.3%) of all patients. Among them, 26 (4.6%) patients were defined as IVIG resistance and 149 (26.2%) patients were considered to have CALs. Patients with missing feeding practice information tended to be older (median age: 18.0 vs 41.0 months, *P* < 0.001).

Two hundred and twenty-three (39.3%) patients were partially breastfed, 293 (51.6%) were exclusively breastfed while 52 (9.2%) were formula-fed. There was a significant difference in C-reactive protein (CRP) among the three groups (*P* < 0.05), although after post hoc test no significance was found between any two groups (formula breastfeeding vs. partial breastfeeding, *P* > 0.9, formula breastfeeding vs. exclusive breastfeeding, *P* = 0.19, partial breastfeeding vs. exclusive breastfeeding, *P* = 0.08). No significant differences were found in any other characteristics (Table 1).

**Table 1:** Characteristics in Kawasaki disease patients with different feeding practices

	Exclusive breastfeeding	Partial breastfeeding	Formula feeding	*P*
N (%)	293 (51.6)	223 (39.3)	52 (9.2)	-
Male, n (%)	179 (61.1)	142 (63.7)	34 (65.4)	0.754
Age of disease onset, months	19.0 (11.0–33.0)	18.0 (11.0–29.0)	15.0 (9.7–25.8)	0.544
Age > 6 months, n (%)	257 (87.7)	202 (90.6)	49 (94.2)	0.871
iKD, n (%)	224 (76.5)	177 (79.4)	46 (88.5)	0.142
IVIG resistance, n (%)	16 (5.5)	7 (3.1)	3 (5.8)	0.417
Delayed IVIG treatment, n (%)	18 (6.1)	20 (9.0)	2 (3.8)	0.296
WBC, × 10^9^/L	14.4 ± 5.4	15.1 ± 5.3	15.9 ± 8.2	0.109
C-reactive protein, mg/dl	57.5 (33.0–93.1)	69.1 (40.9–101.9)	73.4 (44.8–98.6)	0.037
Hemoglobin, g/L	108.1 ± 9.7	108.8 ± 10.4	111.2 ± 8.7	0.141
Hematocrit	0.33 ± 0.03	0.33 ± 0.03	0.33 ± 0.03	0.917
Platelet, × 10^9^/L	353 (289–432)	369 (280–440)	362 (294–425)	0.951
Albumin, g/L	38.9 ± 3.7	39.1 ± 3.8	39.2 ± 3.8	0.819
AST, U/L	32.0 (26.0–47.1)	31.1 (25.2–43.0)	34.4 (28.1–50.7)	0.141
ALT, U/L	24.2 (14.4–63.7)	25.3 (14.8–63.7)	31.0 (18.5–105.7)	0.110
Total bilirubin, umol/L	5.5 (3.9–9.0)	5.3 (3.6–7.9)	5.2 (3.9–8.7)	0.281
LDH, U/L	421.8 ± 121.3	414.1 ± 115.9	442.3 ± 116.0	0.298
Cholesterol, mmol/L	3.6 ± 1.1	3.6 ± 0.8	3.5 ± 0.7	0.632
Creatine kinase, U/L	55.9 (39.8–83.2)	50.8 (36.4–75.8)	53.2 (38.3–76.6)	0.241
Procalcitonin, ng/ml	0.5 (0.2–1.7)	0.6 (0.2–1.5)	0.5 (0.2–2.2)	0.791
Serum sodium, mmol/L	134.6 ± 2.8	134.7 ± 2.8	134.5 ± 2.4	0.896
CALs, n (%)	84 (28.7)	57 (25.6)	8 (15.4)	0.128

Data are expressed as mean ± standard, median (quartiles) or numbers with percentages as appropriate

*iKD* Incomplete Kawasaki disease, *IVIG* Intravenous immunoglobulin, *WBC* White blood cells, *AST* Aspartate aminotransferase, *ALT* Alanine aminotransferase, *LDH* Lactic dehydrogenase, *CALs* Coronary artery lesions

The univariate analysis results by Log-rank test are shown in Table 2. Patients with CALs tended to be infants, and have higher levels of WBC, and platelet count, and have lower levels of hemoglobin, hematocrit, and albumin (*P* < 0.05). Besides, they tended to be iKD and have a lower level of ALT, although the differences were not statistically significant.

**Table 2:** Univariate analysis for coronary artery lesions

	Mean ± SD/median (quartile)/n (%)	CALs, OR (95% CI), *P*
Sex, n (%)		
Male	355 (62.5)	1.0
Female	213 (37.5)	0.79 (0.56, 1.11) 0.179
Age < 12 months, n (%)	153 (26.9)	2.08 (1.40, 3.11) < 0.001
Feeding practice, n (%)		
Formula feeding	52 (9.2)	1.0
Partial breastfeeding	223 (39.3)	1.64 (0.78, 3.43) 0.193
Exclusive breastfeeding	293 (51.6)	1.73 (0.84, 3.57) 0.140
Parity, n (%)		
1	301 (53.1)	1.0
2	239 (42.2)	1.28 (0.92, 1.77) 0.144
3	18 (3.2)	0.22 (0.03, 1.55) 0.127
4	6 (1.1)	1.68 (0.41, 6.86) 0.470
5	3 (0.5)	1.88 (0.26, 13.51) 0.532
Singleton, n (%)		
Yes	551 (97.2)	1.0
No	16 (2.8)	0.70 (0.22, 2.21) 0.546
Prematurity, n (%)		
No	543 (95.9)	1.0
Yes	23 (4.1)	0.32 (0.08, 1.30) 0.113
Delivery methods, n (%)		
Vaginal	334 (59.0)	1.0
Cesarean	232 (41.0)	1.00 (0.72, 1.38) 0.980
IVIG resistance, n (%)		
No	542 (95.4)	1.0
Yes	26 (4.6)	0.53 (0.22, 1.29) 0.161
Delayed IVIG treatment, n (%)		
No	528 (93.0)	1.0
Yes	40 (7.0)	1.50 (0.89, 2.52) 0.126
iKD, n (%)		
No	447 (78.7)	1.0
Yes	121 (21.3)	1.43 (0.99, 2.07) 0.057
WBC, × 10^9^/L	14.8 ± 5.7	1.04 (1.01, 1.07) 0.007
C-reactive protein, mg/dl	62.5 (36.4–98.0)	1.00 (1.00, 1.00) 0.334
Hemoglobin, g/L	108.8 ± 9.7	0.93 (0.91, 0.94) < 0.001
Hematocrit (× 10)	3.3 ± 0.3	0.19 (0.10, 0.34) < 0.001
Platelet, × 10^9^/L	359.0 (288.0–433.5)	1.002 (1.002, 1.003) < 0.001
Albumin, g/L	39.0 ± 3.7	0.94 (0.90, 0.98) 0.002
AST, U/L	31.9 (26.2–46.1)	1.00 (1.00, 1.00) 0.292
ALT, U/L	25.4 (15.0–67.7)	1.00 (1.00, 1.00) 0.058
Total bilirubin, umol/L	5.4 (3.8–8.5)	0.98 (0.96, 1.00) 0.084
LDH, U/L	420.4 ± 118.6	1.00 (1.00, 1.00) 0.908
Cholesterol, mmol/L	3.6 ± 0.9	0.95 (0.80, 1.12) 0.539
Creatine kinase, U/L	54.0 (38.5–77.5)	1.00 (0.99, 1.00) 0.460
Procalcitonin, ng/ml	0.5 (0.2–1.6)	0.97 (0.93, 1.02) 0.288
Serum sodium, mmol/L	134.6 ± 2.8	1.03 (0.97, 1.09) 0.330

Data are expressed as mean ± standard or numbers with percentages as appropriate. Hematocrit was multiplied by 10

*OR* Odds ratio, *CI* Confidence interval, *iKD* Incomplete Kawasaki disease, *IVIG* Intravenous immunoglobulin, *WBC* White blood cells, *AST* Aspartate aminotransferase, *ALT* Alanine aminotransferase, *LDH* Lactic dehydrogenase, *CALs* Coronary artery lesions

Singleton, prematurity, WBC, hematocrit, platelet, iKD, and ALT were selected as the confounders. Three Cox regression models were then established. With formula feeding as a reference, patients who were exclusively breastfed and partially breastfed seemed to have higher incidences of CALs. However, the differences were not significant in all of the three models (Table 3). *P* for trend in these models were insignificant.

**Table 3:** Feeding practices and CALs among patients with Kawasaki disease

	CALs, n (%)	HR (95% CI) *P*		
		Model 1	Model 2	Model 3
Formula feeding	8 (15.4)	1.0	1.0	1.0
Partial breastfeeding	57 (25.6)	1.64 (0.78, 3.43) 0.193	1.48 (0.70, 3.11) 0.305	1.33 (0.63, 2.82) 0.458
Exclusive breastfeeding	84 (28.7)	1.73 (0.84, 3.57) 0.140	1.65 (0.80, 3.42) 0.177	1.51 (0.73, 3.14) 0.268
*P* for trend	0.103	0.215	0.184	0.228

Model 1. No adjustment. Model 2. Adjusted for sex, delayed IVIG treatment, iKD, and IVIG resistance. Model 3. Adjusted for the confounders in Model 2, plus singleton, prematurity, white blood cells, hematocrit, platelet, and alanine aminotransferase

*CALs* coronary artery lesions, *CI* confidence interval, *HR* Hazard ratio

Subgroup analysis was carried out and even after adjusting all covariates (model 3), no significant difference was found in all three age groups (see Additional file 2).

We further tried to seek the effects of the length of breastfeeding on CALs. A total of 508 (89.4%) patients were older than six months and were enrolled in the analysis. Forty-nine patients (9.6%) were formula fed, 26 (5.1%) were breastfed for < 2 months, 56 (11.0%) were breastfed for ≥ 2 and < 4 months, 119 (23.4%) were breastfed for ≥ 4 and < 6 months, 258 (50.8%) were exclusively breastfed for the first 6 months in their lives. Incidence of CALs showed no significant differences among these groups, even after adjusting the potential confounders (*P* > 0.05, see Table 4). Also, *P* for trend in these models were not significant.

**Table 4:** Breastfeeding duration and CALs among patients with Kawasaki disease

	CALs, n (%)	HR (95% CI) *P*		
		Model 1	Model 2	Model 3
Never (formula feeding)	7 (14.3)	1.0	1.0	1.0
< 2 months	7 (26.9)	2.19 (0.76, 6.27) 0.144	1.97 (0.68, 5.68) 0.212	1.59 (0.55, 4.60) 0.392
≥ 2 and < 4 months	11 (19.6)	1.13 (0.44, 2.92) 0.800	1.08 (0.42, 2.81) 0.872	1.06 (0.41, 2.77) 0.899
≥ 4 and < 6 months	30 (25.2)	1.70 (0.74, 3.87) 0.208	1.65 (0.72, 3.79) 0.235	1.42 (0.61, 3.30) 0.417
≥ 6 months	64 (24.8)	1.45 (0.66, 3.17) 0.352	1.43 (0.65, 3.13) 0.372	1.27 (0.58, 2.78) 0.556
*P* for trend	0.117	0.571	0.519	0.713

Only patients older than six months were included (*n* = 508)

Model 1. No adjustment; Model 2. Adjusted for sex, delayed IVIG treatment, iKD, and IVIG resistance. Model 3. Adjusted for the confounders in Model 2, plus singleton, prematurity, white blood cells, hematocrit, platelet, and alanine aminotransferase

*CALs* Coronary artery lesions, *CI* Confidence interval, *HR* Hazard ratio

We included 397 and 359 patients with complete medical data respectively, to carry out the sensitivity analyses, and found the same results (see Additional file 3).

## Discussion

In the present study, we did a retrospective study and tried to investigate the effects of feeding practices and breastfeeding duration on CALs. We found that breastfeeding had no protective effects on the occurrences of CALs in all age groups. Besides, breastfeeding duration was neither associated with CALs.

Breastmilk contains many active immune factors such as secretory immunoglobulin A, oligosaccharides, lactoferrin, nucleotides that have potential protective effects on infectious diseases [11], obesity [12], autoimmune diseases [4, 5], allergic diseases [4], et al. Actually, breastfeeding could potentially reduce hospital admissions for any cause excluding injuries [7]. Thus, breastfeeding has become the preferred method for infant nutrition intake and 90.8% of patients had ever been breastfed in the present study.

There was a significant difference in CRP level, although no difference was found after post hoc tests, seemingly reflecting a protective role of breastfeeding in the disease inflammation. CRP elevation was frequently seen in both infection and inflammation. It was recognized as an important biomarker in the diagnosis of suspected iKD [1] and moreover, was considered as a risk factor for CALs and IVIG resistance in previous studies [13–16]. Besides, no other differences were found regarding ages of disease onset, the subtypes of KD (iKD or complete KD), responses to initial IVIG treatment, or other inflammatory parameters among the three groups.

It seemed that exclusive breastfeeding and partial breastfeeding had no benefit for CALs. Our results were in consistent with a case–control study which was conducted in Central China [9]. Given that breastfeeding practices had no protective effects on CALs, we further explored if the length of breastfeeding played a role. Unfortunately, the results remained the same. In a previous study from Germany, the authors found no difference in CALs with respect to the duration of breastfeeding when they divided breastfeeding duration into longer and shorter than two weeks [8]. However, in a recent study carried out in Taiwan, the authors found that patients who were breastfed for longer than six months had lower occurrences of persisting CALs than those who were breastfed for shorter than six months in univariable analysis. After adjusting the confounders, the difference turned insignificant [17]. Despite infants are better to be exclusively breastfed until 6 months old, and be breastfed until two years old as recommended [18], there are no potential benefits of breastfeeding practice and prolonged breastfeeding regarding the occurrences of CALs in KD in a series of studies. Thus, we could speculate that although exclusive breastfeeding, especially colostrum had a potential role in the prevention of KD onset [7–9], inflammation in patients with CALs was so severe that the protective effects of breastmilk on KD development were partly counteracted. The potential roles of other risk factors for CALs should be explored.

CALs are good indicators of KD severity because they are the main complications of the disease. A higher incidence of CALs was noted in our study than those in Japan and Korea’s national study [19–21]. We largely attributed the divergence to the definition of CALs. The definition of CALs in the two studies [19, 20] was based on the Japanese criteria. In Kim SH’s study [21], the authors reported 24.2% (1666/6889) CALs based on Dallaire’s method, which was also slightly lower than that in our study. It might partly be because we defined patients with clearly irregular appearance of the intima as CALs regardless z scores of the coronary arteries, whereas the luminal irregularity was absent in their study. The cascaded inflammation by immune cells is activated during the acute stage, consequently causing coronary arterial endothelial dysfunction and finally leading to CALs. Although the specific mechanisms are not fully understood, it is generally considered that T cells play important roles in the disease process [22, 23]. Breastmilk is well characterized to be helpful to establish infants’ mature immune systems including T cells [24, 25]. Apart from the reason mentioned above, the reason why breastfeeding has no protective effect likely lies in these aspects, such as genetic backgrounds, meteorological factors, et.al.

It should be noted that a small proportion of patients were IVIG resistant in our study. Previously, we have observed that IVIG resistance rate was only 4.7% during 2006 and 2014 in this area [26]. It was reported that patients who received earlier IVIG treatment than the 4th day were easier to be IVIG resistant [15]. Although IVIG was administered immediately after the diagnosis of KD since 2017 in our hospital as recommended by the latest guidelines [1], there was no significant increase in IVIG non-responders overall.

The present study had some limitations. First, telephone interviews were carried out to obtain the feeding practices of patients, which might cause bias because it was retrospective. Second, we only included KD patients hospitalized in an 18-month period, because the height of patients before May 2017 was not documented. We calculated the z score of each coronary artery rather than the Japanese criteria to better avoid underestimation of CALs in younger patients, especially in infants. However, in the lack of Chinese-originated z score, we used z score equations based on the normal values in the Canadian which might also cause bias. Third, it was reported that maternal factors were also associated with the outcomes of KD [27]. Unfortunately, we were unable to obtain these because they were not routinely recorded on the medical records.

## Conclusion

Although it is reported that breastfeeding lowers the incidence of KD, our results indicate that breastfeeding practice and longer breastfeeding duration couldn’t reduce CALs in those patients who have already developed KD.

## Supplementary Information

**Additional file 1.** Missing number (%) for included variables in dataset.

**Additional file 2.** Subgroup analysis by ages for the associations between feeding practices and CALs.

**Additional file 3.** Cox regression models of feeding practices, breastfeeding duration and CALs among patients with Kawasaki disease.

## Data Availability

The datasets used and/or analysed during the current study are available from the corresponding author on reasonable request.

## Abbreviations

ALT: Alanine aminotransferase
CALs: Coronary artery lesions
CRP: C-reactive protein
HR: Hazard ratio
IVIG: Intravenous immunoglobulin
KD: Kawasaki disease
SD: Standard deviation
VIF: Variance inflation factor
WBC: White blood cells
