# Supplementary material for: Breastfeeding has no protective effects on the development of coronary artery lesions in Kawasaki disease: a retrospective cohort study
Source: BMC Pediatr. 2022 Jun 20;22:353. doi: 10.1186/s12887-022-03422-y (PMC9208131; doi:10.1186/s12887-022-03422-y)
Supplement: Supplementary file 1 — Additional file 1. Missing number (%) for included variables in dataset. [file 12887_2022_3422_MOESM1_ESM.docx]

Additional file 1. Missing number (%) for included variables in dataset

| Variables | Missing, n (%) |
| --- | --- |
| Parity | 1 (0.2) |
| Singleton or multiple births | 1 (0.2) |
| preterm birth | 2 (0.3) |
| White blood cells | 0 (0) |
| C-reactive protein | 4 (0.7) |
| Hemoglobin | 58 (10.2) |
| Hematocrit | 105 (18.5) |
| Platelet | 30 (5.3) |
| Albumin | 4 (0.7) |
| Aspartate aminotransferase | 4 (0.7) |
| Alanine aminotransferase | 4 (0.7) |
| Total bilirubin | 6 (1.0) |
| Lactic dehydrogenase | 4 (0.7) |
| Cholesterol | 6 (1.0) |
| Creatine kinase | 6 (1.0) |
| Troponin I | 154 (27.1) |
| Creatine kinase MB mass | 122 (21.4) |
| Procalcitonin | 49 (8.6) |
| Serum sodium | 39 (6.9) |
