# Supplementary material for: Breastfeeding has no protective effects on the development of coronary artery lesions in Kawasaki disease: a retrospective cohort study
Source: BMC Pediatr. 2022 Jun 20;22:353. doi: 10.1186/s12887-022-03422-y (PMC9208131; doi:10.1186/s12887-022-03422-y)
Supplement: Supplementary file 2 — Additional file 2. Subgroup analysis by ages for the associations between feeding practices and CALs. [file 12887_2022_3422_MOESM2_ESM.docx]

Additional file2. Subgroup analysis by ages for the associations between feeding practices and CALs

|  | HR (95% CI) *P* | | |
| --- | --- | --- | --- |
|  | < 1 yr | ≥ 1 and < 3 yrs | ≥ 3yrs |
| Formula feeding | 1.0 | 1.0 | 1.0 |
| Partial breastfeeding | 1.28 (0.42, 3.85) 0.662 | 1.42 (0.43, 4.71) 0.567 | 0.45 (0.03, 6.89) 0.567 |
| Exclusive breastfeeding | 1.46 (0.49, 4.35) 0.499 | 1.59 (0.48, 5.21) 0.444 | 1.22 (0.14, 10.50) 0.856 |
| *P* for trend | 0.451 | 0.435 | 0.430 |

*CALs* coronary artery lesions, *CI* confidence interval, *HR* Hazard ratio.

All were adjusted for ex, delayed IVIG treatment, iKD, and IVIG resistance, singleton, prematurity, white blood cells, hematocrit, platelet, and alanine aminotransferase.
