# Supplementary material for: Breastfeeding has no protective effects on the development of coronary artery lesions in Kawasaki disease: a retrospective cohort study
Source: BMC Pediatr. 2022 Jun 20;22:353. doi: 10.1186/s12887-022-03422-y (PMC9208131; doi:10.1186/s12887-022-03422-y)
Supplement: Supplementary file 3 — Additional file 3. Cox regression models of feeding practices, breastfeeding duration and CALs among patients with Kawasaki disease. [file 12887_2022_3422_MOESM3_ESM.docx]

Additional file 3. Cox regression models of feeding practices, breastfeeding duration and CALs among patients with Kawasaki disease

|  | CALs, n (%) | HR (95% CI) *P* | | |
| --- | --- | --- | --- | --- |
|  |  | Model 1 | Model 2 | Model 3 |
| Feeding practice |  |  |  |  |
| Formula feeding | 5 (13.9) | 1.0 | 1.0 | 1.0 |
| Partial breastfeeding | 39 (25.3) | 1.86 (0.73, 4.72) 0.193 | 1.74 (0.68, 4.44) 0.245 | 1.52 (0.59, 3.91) 0.383 |
| Exclusive breastfeeding | 59 (28.5) | 1.89 (0.76, 4.72) 0.171 | 1.84 (0.74, 4.58) 0.193 | 1.56 (0.62, 3.91) 0.347 |
| Breastfeeding duration* |  |  |  |  |
| Never (formula feeding) | 4 (11.4) | 1.0 | 1.0 | 1.0 |
| < 2 months | 6 (33.3) | 3.68 (1.04, 13.09) 0.044 | 3.48 (0.97, 12.49) 0.06 | 2.28 (0.62, 8.40) 0.214 |
| ≥ 2 and < 4 months | 6 (14.6) | 1.03 (0.29, 3.65) 0.966 | 0.98 (0.28, 3.50) 0.978 | 0.85 (0.24, 3.05) 0.804 |
| ≥ 4 and < 6 months | 23 (28.4) | 2.52 (0.87, 7.28) 0.089 | 2.45 (0.84, 7.13) 0.010 | 2.19 (0.75, 6.42) 0.154 |
| ≥ 6 months | 48 (26.1) | 1.86 (0.67, 5.16) 0.233 | 1.84 (0.66, 5.12) 0.242 | 1.48 (0.53, 4.15) 0.452 |

*Only patients older than six months were included (n = 359).

*CALs* coronary artery lesions, *CI* confidence interval, *HR* Hazard ratio.

Model 1. No adjustment; Model 2. Adjusted for sex, delayed IVIG treatment, iKD, and IVIG resistance. Model 3. Adjusted for the confounders in Model 2, plus singleton, prematurity, white blood cells, hematocrit, platelet, and alanine aminotransferase.
